# Double-Stranded DNA and NETs Components in Relation to Clinical Outcome After ST-Elevation Myocardial Infarction

**DOI:** 10.1038/s41598-020-61971-7

**Published:** 2020-03-19

**Authors:** Miriam Sjåstad Langseth, Ragnhild Helseth, Vibeke Ritschel, Charlotte Holst Hansen, Geir Øystein Andersen, Jan Eritsland, Sigrun Halvorsen, Morten Wang Fagerland, Svein Solheim, Harald Arnesen, Ingebjørg Seljeflot, Trine Baur Opstad

**Affiliations:** 1 Center for Clinical Heart Research, Oslo University Hospital Ullevål, PB 4956 Nydalen, 0424 Oslo, Norway; 2 Faculty of Medicine, University of Oslo, PB 1078 Blindern, 0316 Oslo, Norway; 3 Department of Cardiology, Oslo University Hospital Ullevål, PB 4956 Nydalen, 0424 Oslo, Norway; 4 Oslo Centre for Biostatistics and Epidemiology, Research Support Services, Oslo University Hospital, PB 4950 Nydalen, 0424 Oslo, Norway

**Keywords:** Coagulation system, Inflammation, Innate immune cells, Biomarkers, Cardiology

## Abstract

Neutrophil extracellular traps (NETs) have been implicated in atherothrombosis; however, their potential role as markers of risk is unclear. We investigated whether circulating NETs-related components associated with clinical outcome and hypercoagulability in ST-elevation myocardial infarction (STEMI). In this observational cohort study, STEMI patients admitted for PCI (n = 956) were followed for median 4.6 years, recording 190 events (reinfarction, unscheduled revascularization, stroke, heart failure hospitalization, or death). Serum drawn median 18 hours post-PCI was used to quantify double-stranded DNA (dsDNA) and the more specific NETs markers myeloperoxidase-DNA and citrullinated histone 3. Levels of the NETs markers did not differ significantly between groups with/without a primary composite endpoint. However, patients who died (n = 76) had higher dsDNA compared to survivors (p < 0.001). Above-median dsDNA was associated with an increased number of deaths (54 vs. 22, p < 0.001). dsDNA in the upper quartiles (Q) was associated with increased mortality (Q3 vs. Q1 + 2 adjusted HR: 1.89 [95% CI 1.03 to 3.49], p = 0.041 and Q4 vs. Q1 + 2 adjusted HR: 2.28 [95% CI 1.19 to 4.36], p = 0.013). dsDNA was weakly correlated with D-dimer (*r*_*s*_ = 0.17, p < 0.001). dsDNA levels associated with increased all-cause mortality, yet weakly with hypercoagulability in STEMI patients. The prognostic significance of potentially NETs-related markers requires further exploration.

## Introduction

Acute coronary syndrome (ACS) often results from erosion or rupture of atherosclerotic plaques followed by thrombosis, ischemia, and myocardial injury. It has been suggested that the risk-stratification of ACS patients should be expanded to include new soluble biomarkers, which may help individualize treatment^1^. Potential candidates include components of the innate immune response, widely accepted to be important in the immediate aftermath of ACS, yet still not completely understood.

Neutrophil extracellular traps (NETs) were first described in 2004 by Brinkmann and colleagues, who documented the expulsion of extracellular webbed structures comprising deoxyribonucleic acid (DNA) and histones, studded with neutrophil granule proteins^2^. The process of NETs release, or NETosis, is initiated by granular proteins such as myeloperoxidase (MPO), neutrophil elastase, and peptidylarginine deiminase 4^3^. The latter mediates the citrullination of histone 3, inducing chromatin decondensation before extracellular release^3^.

Circulating NETs-associated components may contribute to endothelial dysfunction and plaque instability^4^, as well as coronary thrombus formation^5^ by activating platelets, triggering the coagulation cascade, and trapping thrombus constituents^6,7^. Nevertheless, it is not yet clear how NETs *per se* are implicated in the fine balance between beneficial and deleterious effects of inflammation after revascularization.

Although circulating double-stranded deoxyribonucleic acid (dsDNA) alone lacks specificity, it has been used as a surrogate NETs-related component due to the reliable, single-step and high-throughput nature of available methods, making it appealing as a putative biomarker^8^. Other components that are regarded as highly specific to NETs release include DNA complexed with neutrophil-derived proteins, such as myeloperoxidase-DNA (MPO-DNA), and citrullinated histone 3 (CitH3)^8,9^.

On the basis that enhanced NETs release may reflect an exaggerated immune response to ischemic myocardial injury, we hypothesized that elevated levels of circulating NETs-related components might indicate poorer prognosis in patients with ACS. We therefore aimed to characterize any association between the levels of dsDNA, the more NETs-specific MPO-DNA and CitH3, and clinical outcome in a population with ST-segment elevation myocardial infarction (STEMI). Secondarily, we assessed any relation to selected markers of hypercoagulability.

## Material and Methods

### Study subjects

Patients with a STEMI diagnosis admitted to Oslo University Hospital Ullevål for percutaneous coronary intervention (PCI) were consecutively and prospectively included in an observational cohort study between 2007 and 2011 (*n* = 1026), as previously described^10^. Patients using oral anticoagulants were excluded prior to analysis due to the potential interaction with NETs and coagulation markers. In total, 956 patients were eligible for the present post hoc sub-study (Supplementary Fig. S1). Hospital records and patient questionnaires were collected for clinical data. Echocardiography was performed during the initial hospital admission or at the patient’s local hospital within three months after the index infarction. Left ventricular ejection fraction (LVEF) measurements were available in 738 patients, performed either by visual approximation or using Simpson’s biplane method. All participants gave written informed consent. The study complies with the Declaration of Helsinki, and was approved by the Regional Ethics Committee of South East Norway (project ID 1.2006.1975).

### Definitions

STEMI was defined as ST-segment elevation of ≥0.2 mV in two or more contiguous chest leads, ≥0.1 mV in two or more limb leads, or new-onset left bundle branch block on electrocardiogram, combined with chest pain or symptoms typical of myocardial ischemia, and troponin levels >99^th^ percentile.

Diabetes mellitus was defined as previously treated diabetes. Hypertension was defined as current use of antihypertensive drugs or previously known diagnosis. Current smoking was defined as regular tobacco smoking or cessation <3 months prior to inclusion. Previous cardiovascular disease (CVD) was defined as a history of myocardial infarction (MI), PCI, coronary artery bypass graft, or stroke.

### Clinical endpoints

Outcomes were censored at the end of 2013, after a median observation time 4.6 years. The primary endpoint was a composite of reinfarction, stroke, rehospitalization for heart failure, unscheduled revascularization >3 months after the index infarction, or all-cause mortality, whichever occurred first. All-cause mortality was a pre-specified secondary endpoint. An endpoint committee reviewed follow-up data acquired from patient contact, medical records, and the Norwegian Cause of Death Registry.

### Laboratory methods

Venous blood samples were collected in a fasting state (8:00–10:00 a.m.), a median of 24 hours after symptom onset and median 18 hours post-PCI. Citrated plasma was kept on ice until centrifugation within 30 min (3000 *g* and 4 °C for 20 min). Serum was centrifuged within an hour of sampling (2500 *g* for 10 min), and stored at −80 °C pending analysis.

Serum double-stranded DNA (dsDNA) levels were quantified using a fluorescent nucleic acid stain, Quant-iT PicoGreen (Invitrogen Ltd., Paisley, UK) and fluorometry (Fluoroskan Ascent fluorometer, Thermo Fisher Scientific, Vantaa, Finland). Myeloperoxidase-deoxyribonucleic acid complexes (MPO-DNA) were measured in serum using an enzyme-linked immunosorbent assay (ELISA) technique as described by Kessenbrock *et al*^11^. In brief, plates were coated with the capture antibody anti-MPO (AbD Serotec, Hercules, CA, USA) overnight at 4 °C. After blocking with BSA, patient serum and a peroxidase-labeled anti-DNA antibody (Cell Death Detection kit, Roche Diagnostics GmbH, Mannheim, Germany) were added and incubated for two hours. Finally, a peroxidase substrate was added and absorbance measured after 40 min, reporting data as optical density (OD) units due to lack of a comparative standard. Citrullinated histone 3 (CitH3) was quantified using a commercial sandwich ELISA kit (Cayman Chemical, Ann Arbor, USA). Samples below the limit of detection were set to zero (*n* = 19). The inter-assay coefficient of variation (CV) for dsDNA, MPO-DNA, and CitH3 were 6.3%, 9.1%, and 12.4%, respectively.

High-sensitivity C-reactive protein (CRP) was quantified with an inter-assay CV < 5% (DRG Instruments, Marburg/Lahn, Germany). N-terminal pro-B-type natriuretic peptide (NT-proBNP) and peak cardiac Troponin T (TnT) were measured in serum using commercial electrochemiluminescence immunoassays (Elecys NT-proBNP, Roche Diagnostics, Indianapolis, USA and third generation cTroponin T, Elecys 2010, Roche, Mannheim, Germany). The inter-assay CV was 7% for both methods.

Plasma levels of prothrombin fragment 1 and 2 (F1+2) and D-dimer were determined by commercially available enzyme immunoassays (Enzygnost F1+2 monoclonal, Siemens, Marburg, Germany, and Asserachrom D-dimer, Stago Diagnostica). The inter-assay CVs were 6.5 and 5.4%, respectively.

### Statistical analysis

**Figure 1:**
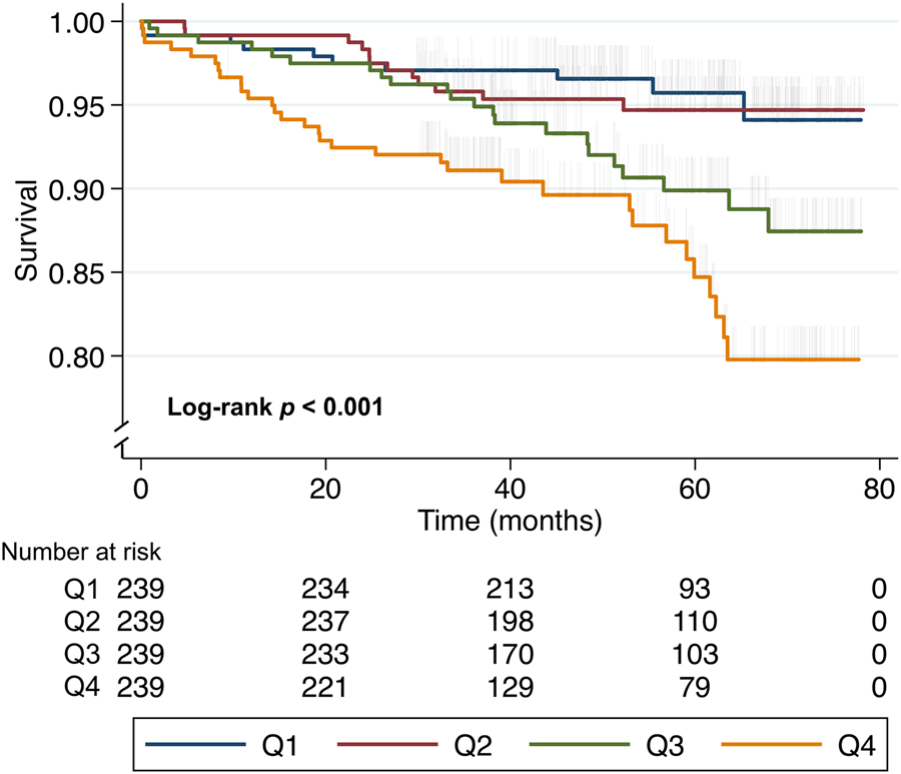
Time-to-event curves (months) for each quartile (Q) of dsDNA according to all-cause mortality. Q1: ≤ 371 ng/ml. Q2: 372–414 ng/ml. Q3: 415–470 ng/ml. Q4: ≥ 471 ng/ml. Vertical lines indicate censoring. The p-value refers to the difference across quartiles, as assessed using the log-rank test. Maximum follow-up time was 78 months (6.5 years). Figure created using software from StataCorp. 2017. *Stata Statistical Software: Release 15*. College Station, TX: StataCorp LLC. Available from https://www.stata.com.

Data are presented as mean (±SD), median (25^th^, 75^th^ percentile), or proportions (%) as appropriate. Bivariate correlation analyses were performed using Spearman’s rho (*r*_*s*_) due to skewed distribution of the NETs markers, and a complete correlation table including 95% confidence intervals (CI) calculated using the Fisher Z transformation is shown in Supplementary Table S1. A Bonferroni correction was applied to Supplementary Table S1 to account for multiple testing. Corrections otherwise have not been performed due to the explorative nature of this study. Normally or skewly distributed continuous data were compared using the unpaired two-sample Student *t*-test and Mann-Whitney U test, respectively. Chi-squared tests were used to compare proportions. Survival curves were produced using Kaplan-Meier methods, and differences in survival distributions estimated by log-rank chi-squared tests. As the two lower quartiles of dsDNA displayed a similar survival distribution (Fig. 1), these were aggregated in subsequent analyses. Crude and adjusted hazard ratios (HRs) were calculated by Cox proportional hazards regression models to assess the impact of dsDNA on mortality risk. Age, sex, and smoking were included in the adjusted model by convention. Other potential confounding variables were considered for inclusion in the adjusted model if there was *both* a significant association with dsDNA levels (Table 1), as well as a significant relation to the dependent variable as assessed by crude Cox regression models (Table 2). Additionally, although not fulfilling these criteria, a second adjusted model is presented in section 3.6 and Supplementary Table S5 to address the potential expansion of the model to include clinically important variables (time, peak TnT, thrombolysis and history of previous CVD). Sensitivity analyses were performed to assess the impact of substituting one confounder variable with a highly correlated variable, such as LVEF ≤ 40% and NT-proBNP, to ensure that they were truly interchangeable in the final adjusted model. The level of statistical significance was set to *p* ≤ 0.05. Analyses were performed using IBM SPSS Statistics v.25 and Stata/SE v.15 software.

**Table 1:** Baseline characteristics of the total study population and according to above- or below-median levels of dsDNA.

	Total study population (*n* = 956)	dsDNA		p*
		<median	≥median	
Age, mean (range)	60.8 (24–94)	61.4 (24–94)	60.1 (29–88)	0.107
Male sex	767 (80.2)	374 (78.2)	393 (82.2)	0.123
Current smoking	450 (47.1)	208 (43.5)	242 (50.6)	**0.028**
Hypertension	326 (34.1)	163 (34.1)	163 (34.1)	1.000
Diabetes	122 (12.8)	61 (12.8)	61 (12.8)	1.000
Previous CVD	228 (23.6)	106 (22.2)	122 (25.6)	0.218
Medication on admission				
- Single or DAPT	222 (23.2)	108 (22.6)	114 (23.8)	0.646
- Statins	215 (22.5)	103 (21.5)	112 (23.4)	0.486
- Beta-blockers	182 (19.0)	84 (17.6)	98 (20.5)	0.249
- ACEi/ARB	234 (24.5)	114 (23.8)	120 (25.1)	0.652
BMI, kg/m^2^	26.6 (24.3, 29.2)	26.3 (24.3, 29.3)	26.9 (24.2, 29.1)	0.549
Leukocyte count, x10^9^/L	10.7 (8.7, 13.3)	10.2 (8.3, 12.5)	11.3 (9.1, 14.1)	**<0.001**
Platelet count, x10^9^/L	221 (188, 265)	213 (183, 263)	225 (195, 266)	**0.017**
Total cholesterol, mmol/L	4.86 ± 1.12	4.89 ± 1.10	4.84 ± 1.13	0.462
LDL-cholesterol, mmol/L	3.24 ± 1.01	3.29 ± 1.01	3.19 ± 1.01	0.123
Fasting glucose, mmol/L	5.8 (5.2, 6.7)	5.7 (5.2, 6.5)	5.9 (5.3, 6.7)	**0.008**
HbA_1c_, %	5.9 (5.6, 6.3)	5.9 (5.6, 6.2)	5.9 (5.6, 6.3)	0.797
NT-proBNP, ng/L	31 (11, 118)	24 (10, 73)	50 (12, 182)	**<0.001**
Peak TnT, ng/L	3850 (1723, 7228)	3405 (1515, 6338)	4635 (2070, 8138)	**<0.001**
CRP, mg/L	13.8 (7.0, 32.2)	11.4 (6.5, 24.0)	16.8 (8.1, 38.8)	**<0.001**
LVEF ≤ 40%	145 (15.2)	52 (14.3)	93 (24.9)	**<0.001**
Prehospital thrombolysis	107 (11.2)	49 (10.3)	58 (12.1)	0.356
Anterior wall infarction	413 (43.2)	193 (40.4)	220 (46.0)	0.078
Time from symptom onset to blood sampling, hours	24 (18, 32)	23 (18, 31)	24 (19, 36)	**0.017**

Values given as mean (±SD) or (range), median (25^th^, 75^th^ percentiles) or numbers (%) as appropriate.

*p-value of Mann-Whitney U, Student’s t, or Chi squared tests comparing groups with below- and above-median levels of dsDNA.

DAPT: dual anti-platelet therapy.

ACEi: angiotensin-converting enzyme inhibitor.

ARB: angiotensin II receptor blocker.

BMI: body mass index.

LDL: low-density lipoprotein HbA1c: hemoglobin A1c.

**Table 2:** Crude/unadjusted and adjusted Cox regression analysis of the association between dsDNA and all-cause mortality. The adjusted multivariable model included age, sex, smoking, leukocyte count, platelet count, NT-proBNP, fasting glucose, CRP and D-dimer as potential covariates.

	*n*	Univariable analysis			Multivariable analysis (*n* = 907)		
		Unadjusted HR	95% CI	p*	Adjusted HR	95% CI	p*
**dsDNA Q3 vs. Q1**+**2**	956	**2.03**	**1.12 to 3.66**	**0.019**	**1.89**	**1.03 to 3.49**	**0.041**
**dsDNA Q4 vs. Q1**+**2**	956	**3.36**	**1.95 to 5.78**	**<0.001**	**2.28**	**1.19 to 4.36**	**0.013**
Age	956	1.09	1.07 to 1.12	**<0.001**	1.10	1.07 to 1.13	**<0.001**
Male vs. female sex	956	0.40	0.25 to 0.64	**<0.001**	0.77	0.45 to 1.32	0.344
Current smoking (+/−)	956	0.87	0.55 to 1.36	0.535	1.89	1.11 to 3.24	**0.020**
Leukocyte count	953	1.03	1.00 to 1.06	**0.025**	0.94	0.87 to 1.02	0.136
Platelet count	952	1.01	1.00 to 1.01	**<0.001**	1.01	1.00 to 1.01	**0.001**
NT-proBNP	926	1.00	1.00 to 1.00	**<0.001**	1.00	1.00 to 1.00	**0.049**
Fasting glucose	947	1.17	1.05 to 1.29	**0.003**	1.25	1.11 to 1.40	**<0.001**
CRP	955	1.01	1.00 to 1.01	**0.022**	1.00	1.00 to 1.01	0.830
D-dimer	950	1.00	1.00 to 1.00	**<0.001**	1.00	1.00 to 1.00	0.051
Previous CVD (+/−)	955	2.25	1.42 to 3.56	**0.001**			
Peak TnT	956	1.03	0.99 to 1.07	0.150			
Prehospital thrombolysis (+/−)	956	0.21	0.05 to 0.86	**0.030**			
Time (symptom onset to blood sampling)	951	1.00	0.99 to 1.01	0.722			

Hazard ratios per one unit increase in the explanatory variables, except where otherwise stated, e.g. presence/absence of categorical variables (+/−). *p-value corresponding to Wald test.

## Results

### Study population

Baseline characteristics of the study population (*n* = 956), as well as according to groups with above- and below-median levels of dsDNA are shown in Table 1. Characteristics according to above- and below-median levels of MPO-DNA and CitH3 are displayed in Supplementary Table S2. The mean age was 61 years and 80% were men. Previously diagnosed CVD was recorded in 23.6% of patients, whereas only 2.3% had undergone a coronary artery bypass graft operation. Nearly all patients were treated with acetylsalicylic acid, clopidogrel and heparin (98–100%) according to standard of care prior to PCI, and 35% of patients received glycoprotein IIb/IIIa inhibitors peri-procedurally. As to choice of coronary stent, 77% of patients received a bare metal stent while 17% received a drug-eluting stent. One percent received both stent types, whereas in 5% no stents were employed.

### NETs-related components and leukocyte count

The NETs-related components were all skewly distributed, with median values: dsDNA 414 ng/ml (372, 470), MPO-DNA 0.177 OD (0.139, 0.258), and CitH3 9.21 ng/ml (4.87, 17.24). They all inter-correlated moderately (*r*_*s*_ = 0.29–0.36, p < 0.001 for all) (Supplementary Fig. S2, Supplementary Table S1). Moreover, dsDNA, MPO-DNA and CitH3 correlated weakly with total leukocyte count (*r*_*s*_ = 0.22, 0.18, and 0.09, respectively, p ≤ 0.005 for all).

### NETs-related components and myocardial function

dsDNA and the more NETs-specific MPO-DNA were both weakly correlated with peak TnT (*r*_*s*_ = 0.17 and 0.12, respectively, p < 0.001 for both, Supplementary Table S1), whereas CitH3 was not. Correspondingly, levels of both dsDNA and MPO-DNA were significantly higher among patients suffering an anterior MI (43.2%) as compared to other infarct locations (424 ng/ml (376, 476) vs. 409 ng/ml (369, 465) and 0.188 OD (0.146, 0.292) vs. 0.171 OD (0.135, 0.244), *p* ≤ 0.031 for both). A similar, but non-significant difference was observed for CitH3 levels (9.71 ng/ml (5.19, 17.27) vs. 8.69 ng/ml (4.61, 17.13), *p* = 0.094). Levels of the NETs-related components were not associated with type of stent inserted during PCI (data not shown).

dsDNA and CitH3 levels were weakly correlated with NT-proBNP levels (*r*_*s*_ = 0.10–0.19, p ≤ 0.001 for both, Supplementary Table S1), whereas MPO-DNA was not. Patients with a reduced EF ≤ 40% also had significantly elevated levels of both dsDNA and MPO-DNA (439 ng/ml (398, 491) vs. 409 ng/ml (366, 464) and 0.196 OD (0.148, 0.312) vs. 0.175 OD (0.137, 0.254), respectively, *p* ≤ 0.007 for both).

### NETs-related components and hypercoagulability

dsDNA levels were weakly correlated with D-dimer (*r*_*s*_ = 0.17, p < 0.001, Supplementary Table S1). Dichotomising dsDNA at the median, above-median levels were significantly associated with elevated D-dimer (538 ng/ml (305, 952) vs. 415 ng/ml (279, 724), *p* < 0.001) and F1+2 (260 pmol/L (182, 575) vs. 234 pmol/L (176, 332), *p* = 0.01) (Supplementary Fig. S3). No significant correlations to D-dimer or F1+2 were encountered for MPO-DNA or CitH3. None of the NETs-related components were associated with the administration of glycoprotein IIb/IIIa inhibitors (data not shown) or prehospital thrombolysis (Table 1 and Supplementary Table S3).

### NETs-related components and clinical endpoints

In total, 190 patients (19.9%) experienced a clinical event (60 deaths, 58 reinfarctions, 51 urgent revascularizations, 6 strokes, and 15 hospitalizations due to decompensated heart failure). A total of 76 patients (7.9%) died during follow-up, including 16 deaths occurring secondary to a primary endpoint. Median times to first clinical event and death of any cause were 17 and 26 months after the index infarction, respectively.

Baseline characteristics stratified according to groups experiencing a primary composite endpoint and all-cause mortality are presented in Supplementary Table S3. Patients experiencing a clinical event were significantly older, more likely to have a history of CVD, and less likely to have received prehospital thrombolysis. Among patients experiencing a primary composite endpoint, a higher proportion used statins, antiplatelet and antihypertensive drugs on admission for the index infarction. These patients also had significantly elevated fasting glucose and NT-proBNP levels, and correspondingly were more likely to have a reduced LVEF ≤ 40%.

**Figure 2:**
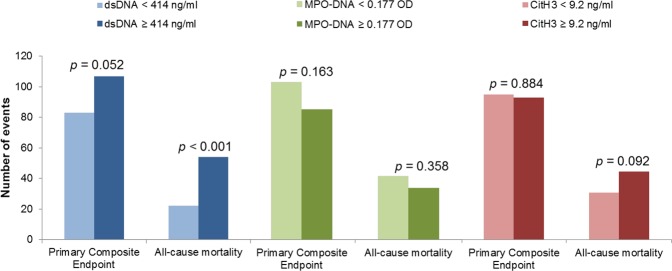
Number of primary composite endpoints and deaths according to above- or below-median levels of dsDNA (blue), MPO-DNA (green), and CitH3 (red). The p-values refer to Chi-square tests. Figure created using software from IBM Corp. Released 2017. IBM SPSS Statistics for Windows, Version 25.0. Armonk, NY: IBM Corp. Available from https://www.ibm.com/se-en/products/spss-statistics.

None of the NETs-related components differed significantly between groups with or without a primary composite endpoint (Supplementary Table S4). When exploring the endpoints occurring in the first one, 6, 12, or 24 months separately (*n* = 20, 50, 75, and 117, respectively), these findings remained unchanged. There were no significant between-group differences in number of primary endpoints when NETs components were dichotomised at the medians, although a non-significant difference in clinical event-rate was observed for above-median levels dsDNA (107 vs. 83 events, *p* = 0.052) (Fig. 2).

Among the 76 patients who died during follow-up, dsDNA levels were significantly elevated (460 ng/ml (407, 508) vs. 411 ng/ml (370, 466), *p* < 0.001) (Supplementary Table S4). Patients with above-median dsDNA levels had a significantly higher all-cause mortality rate (54 vs. 22, *p* < 0.001) (Fig. 2). No significant associations to all-cause mortality were observed for MPO-DNA or CitH3.

### dsDNA levels and survival

There were significant differences in survival across quartiles of the putative NETs-related component dsDNA (log-rank *p* < 0.001) (Fig. 1). Patients with dsDNA levels in the third and fourth quartile (Q3 and Q4), had a two- and three-fold risk of death compared to the lowest two quartiles combined (Q1+2) (Table 2).

After adjusting for potential confounders, patients with dsDNA levels in Q3 and Q4 versus Q1+2 still had a significantly increased mortality rate (HR 1.89 [95% CI 1.03 to 3.49] and HR 2.28 [95% CI 1.19 to 4.36], respectively) (Table 2). When adjusting for additional variables of clinical importance, including time elapsed between symptom onset to blood sampling, peak TnT levels, previous CVD, and administration of thrombolysis, dsDNA in the two upper quartiles still conferred an increased risk of death during long-term follow-up (dsDNA Q3 vs. Q1+2: HR 1.88 and Q4 vs. Q1+2: HR 2.06) (Supplementary Table S5). As there were no significant associations between endpoint rates and the more NETs-specific components MPO-DNA or CitH3, regression and survival analyses were not performed for these markers.

## Discussion

The present study is to our knowledge the first report on several circulating NETs-related components in a large cohort of STEMI patients. Neither dsDNA, nor the more NETs-specific markers MPO-DNA and CitH3, were associated with adverse clinical outcome as defined by the primary composite endpoint. However, high levels of dsDNA in the subacute phase after STEMI were associated with an increased risk of death during long-term follow-up, and associated weakly with markers of hypercoagulability. Despite this interesting finding, the relevance of NETs-specific components in the peripheral circulation as biomarkers of risk is still unclear and remains to be further explored.

The NETs-related components were not associated with the occurrence of the primary composite endpoint; however, we observed more frequent composite endpoints with high dsDNA levels. Though potentially also reflecting other non-NETosis processes, cell-free DNA has previously been linked to complications after ACS including cardiac arrest, heart failure, rehospitalization, and mortality during two-year follow-up^12^, and was shown to correlate with Global Registry of Acute Coronary Events scores in a STEMI cohort^13^. In our population, peripheral levels of the more NETs-specific components CitH3 and MPO-DNA did not reflect risk of clinical events, even though MPO alone has previously been described as a strong predictor of cardiovascular events after ACS^14^. The aspect of follow-up time could be of importance, as higher MPO levels have been reported to associate with 30-day risk of recurrent ischemic events, yet this risk was attenuated by 6 months^15^. However, we could not show any short-term association with the primary composite endpoint either. Exploring the idea of ‘biological compartments’, Mangold *et al*. found that markers of neutrophil degranulation and NETs release were significantly higher at the culprit lesion site as compared to femoral blood samples^5^. Other data showed that the levels of NETs components were significantly elevated only in the infarct-related artery, but not in other coronary arteries when compared to intracoronary samples from normal diagnostic angiographies, supporting compartmentalisation^16^. However, dsDNA in the coronary and peripheral circulation is reportedly highly correlated^17^. Interestingly, it was also recently demonstrated in STEMI patients that even though both dsDNA and the more specific marker MPO-DNA were higher in the coronary than peripheral circulation, a considerable overlap was detected for dsDNA levels but not MPO-DNA levels, suggesting that MPO-DNA measured peripherally is a poorer reflection of coronary NETosis^18^. Thus, it appears that dsDNA and the more specific NETs markers measured in the peripheral circulation differentially represent the local thrombo-inflammatory milieu in the coronary circulation with adhesive stationary neutrophils. This could explain the observed lack of congruence between the three systemically quantified NETs-related components, as well as the lack of association with the primary composite endpoint. The neutral findings with respect to the primary composite endpoint in our study could also be due to the aggregation of endpoints with different underlying pathophysiology.

Higher dsDNA levels were associated with poorer long-term survival after STEMI, even after adjusting for cardiovascular risk factors. This is in accordance with Wang *et al*. who also found significantly higher cardiovascular mortality after a follow-up of two years amongst patients with STEMI and high dsDNA levels^17^. This might be discussed as an effect of an exaggerated immune response to infarction, corroborated by the positive correlation, although weak, between dsDNA and total leukocyte count. Although we cannot confirm the origin of the cell-free DNA from our data, DNA itself could conceivably stimulate both NETs release and the generation of reactive oxygen species that play a central role in ischemia and reperfusion (IR)-injury^19,20^. This idea is, however, not supported by our results with a lack of association between survival and the NETs-specific markers. Higher intracoronary levels of cell-free DNA during PCI, as well as NETs burden in coronary thrombi, have been associated with lack of ST-segment resolution and reperfusion, clinical markers of IR-injury^5,21^. On the other hand, the correlations between both dsDNA and the NETs-specific marker MPO-DNA with peak TnT, a traditional marker of cardiac injury, were weak. We have previously shown that dsDNA correlated with peak TnT and infarct size five days after PCI^22^, also supported by several other studies^5,23,24^. Nonetheless, the lack of a clear association with TnT may indicate that the measured dsDNA has not merely leaked from damaged cardiomyocytes. This is corroborated by our previous observation that dsDNA peaked prior to PCI, indicating the importance of temporality which this study could not assess^25^. Even though causality cannot be established from our observations, and there is incongruence between the NETs-specific components, multivariable analyses show that even after adjusting for variables related to myocardial injury and function such as TnT and NT-proBNP, dsDNA still confers an increased risk of death.

Hypercoagulability is an important determinant of clinical outcome after STEMI^26^. It has been demonstrated that neutrophil-platelet aggregates and NETs are an integral part of coronary thrombi^5^, and one might therefore speculate that poorer survival is related to intracoronary thrombotic burden and microvascular occlusions. However, the only observed link with a prothrombotic state in the present study was a weak association between D-dimer and dsDNA. Again, this could be discussed as a result of localised tissue factor activity in the coronary circulation, enabling concentrated NETs release and thrombosis without a significant effect on the systemic circulation. A “two-hit hypothesis”, where both an inflammatory stimulus and activated platelets are required for NETosis in STEMI could explain the lack of association between the NETs-specific components and hypercoagulability markers peripherally^16^. We have previously shown that a prothrombotic state was predictive of death in this population, even after adjusting for the administration of prehospital thrombolysis^27^. Although fewer endpoints were recorded among patients receiving thrombolysis, the risk of death conferred by higher dsDNA levels was relatively unchanged when adjusting for both thrombolysis and D-dimer levels. Nonetheless, the diverging findings do not support prothrombotic NETs-specific effects, yet perhaps suggest that circulating dsDNA might be equally important in non-thrombotic ACS processes.

We could not show any significant associations between the NETs-specific components MPO-DNA or CitH3 and mortality. One explanation could be that the MPO and DNA molecules with opposite charges might bind *after* being released into the circulation, independent of NETosis^28^. Myocardial injury could also inhibit NETosis, as discussed in an experimental study showing that although apoptotic cells were a potent stimulus of NETs release, neutrophils could not undergo NETosis after phagocytosing apoptotic cells^29^. Although the intercorrelations between the putative NETs-related components were similar to previous reports^30^, perhaps the discrepancies between the markers could reflect different pathophysiological processes or types of NETosis. The extrusion of chromatin from neutrophils is seemingly a heterogeneous process, and there is no consensus as to which pathways identified *in vitro* are of importance *in vivo*. It has been suggested that citrullination of histone 3 is not universally required for NETosis^31^, potentially explaining our divergent CitH3 observations. Differences in NETs composition depending on the stimulus, have also been described^32,33^. Beyond the discrepancies between the putative NETs markers, it is possible that the markers also represent the *resolution* of inflammation after ischemic injury, and that NETs likely exert both damaging *and* beneficial effects in thrombo-inflammatory conditions such as STEMI. Nonetheless, the present study does not provide evidence for the clinical importance of the NETs components measured peripherally, and further work is warranted to improve the sensitivity and specificity of the markers.

A major limitation of the study is the single blood sampling and timing after symptom onset. Lack of knowledge about the release kinetics and half-life of circulating NETs components complicates interpretation of the results. The timing of the PCI procedure after symptom onset and PCI-related use of unfractionated heparin could also be of significance. Heparin is reported to remove platelet aggregates, almost completely dismantle NETs^6^, and can interfere with cell-cell interactions^34^, potentially attenuating neutrophil activation and NETosis. Second, although it has been shown that circulating dsDNA reflects NETs burden in coronary thrombi^5^, caution is required when assessing NETosis based solely on dsDNA observations. Mitochondrial DNA is also reportedly elevated in ACS patients^35^, and several studies point out that cardiomyocytes contain abundant chromatin, possibly contributing to circulating DNA^13,36,37^. Third, inconsistencies and only moderate intercorrelations between the surrogate NETs-related components, nonetheless comparable to previous reports, complicate data interpretation^22,30^. Fourth, NETs-derived DNA is likely not fragmented as with apoptosis^38^, thus the higher molecular weight and accompanying electrical charges could have interfered with the MPO-DNA assay. It may also be that the use of plasma samples is preferable in quantifying *in vivo* NETs components^39^, potentially reducing interference from serum proteases. Fifth, there was likely some selection bias, as many potentially eligible patients did not provide consent for unknown reasons, potentially impacting external generalizability. Critically ill patients in particular might be unable to give consent, precluding participation. Patients with immune-mediated disease may have been included, resulting in residual confounding for which we have not adjusted. Further, LVEF was recorded at different time points, possibly capturing reversible stunning or hibernation in early measurements. Finally, it would be of interest to further classify cause of death to better understand potential clinical implications of dsDNA as a biomarker. Nor could we assess underlying STEMI aetiologies, such as plaque erosion versus rupture, in which NETs might play differential roles^40,41^.

The present study in STEMI patients reaffirms the association between dsDNA levels and all-cause mortality during long-term follow-up, but could not demonstrate a relationship between NETs-specific components and clinical outcome. The observed weak associations between the less specific marker dsDNA only, and markers of hypercoagulability and myocardial injury, were seemingly independent of associations with overall mortality. These findings seem to confirm the potential clinical relevance of circulating dsDNA in STEMI, yet discrepancies between the NETs-specific components draw into question their prognostic significance in ACS when measured peripherally.

## Supplementary information

Supplementary information.

## Data Availability

Anonymised data can be made available on request.
