# Supplementary material for: Double-Stranded DNA and NETs Components in Relation to Clinical Outcome After ST-Elevation Myocardial Infarction
Source: Sci Rep. 2020 Mar 19;10:5007. doi: 10.1038/s41598-020-61971-7 (PMC7081350; doi:10.1038/s41598-020-61971-7)
Supplement: Supplementary file 1 — Supplementary information. [file 41598_2020_61971_MOESM1_ESM.pdf]

DOUBLE-STRANDED DNA AND NETs COMPONENTS IN RELATION TO CLINICAL OUTCOME AFTER ST-ELEVATION MYOCARDIAL INFARCTION

SUPPLEMENTARY MATERIAL

Miriam Sjåstad Langseth* [1, 2], MD, Ragnhild Helseth [1, 2], MD PhD, Vibeke Ritschel [1, 2], MD PhD, Charlotte Holst Hansen [1, 3], MN, Geir Øystein Andersen [1, 3], MD PhD, Jan Eritsland [1, 3], MD PhD, Sigrun Halvorsen [2, 3], Professor MD PhD, Morten Wang Fagerland [4], MSc PhD, Svein Solheim [1, 3], MD PhD, Harald Arnesen [1, 2], Professor emeritus, MD PhD, Ingebjørg Seljeflot [1, 2, 3], Professor PhD, Trine Baur Opstad [1, 2], MSc PhD.

[1] Center for Clinical Heart Research, Oslo University Hospital Ullevål, PB 4956 Nydalen, 0424 Oslo, Norway
[2] Faculty of Medicine, University of Oslo, PB 1078 Blindern, 0316 Oslo, Norway
[3] Department of Cardiology, Oslo University Hospital Ullevål, PB 4956 Nydalen, 0424 Oslo, Norway
[4] Oslo Centre for Biostatistics and Epidemiology, Research Support Services, Oslo University Hospital, PB 4950 Nydalen, 0424 Oslo, Norway

Corresponding author: Miriam Sjåstad Langseth
E-mail address: m.s.langseth@studmed.uio.no
Telephone number: +47 993 87 261
Address: Center for Clinical Heart Research, Oslo University Hospital Ullevål,
PB 4956 Nydalen, 0424 Oslo, Norway

Keywords: Neutrophil extracellular traps (NETs), atherothrombosis, acute coronary syndrome (ACS), ST-elevation myocardial infarction (STEMI)

Reference: Figures S2 and S3 were created using software from IBM Corp. Released 2017. IBM SPSS Statistics for Windows, Version 25.0. Armonk, NY: IBM Corp. Available from https://www.ibm.com/se-en/products/spss-statistics.

Figure S1. Study flow chart.

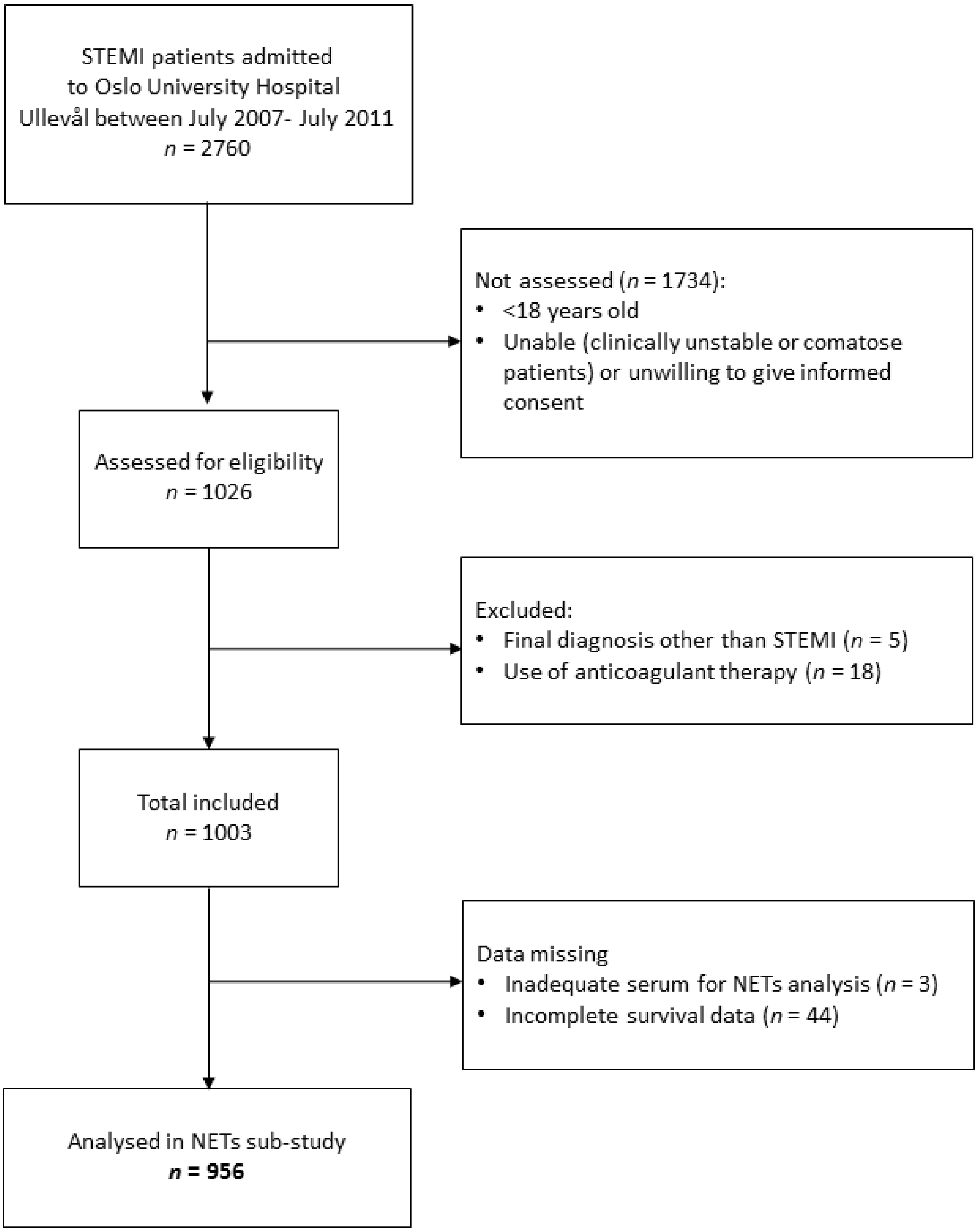

Figure S2. Scatter-plots demonstrating the relationship between potential NETs-related markers.

The *r*- and *p*-values refer to Spearman's rho.

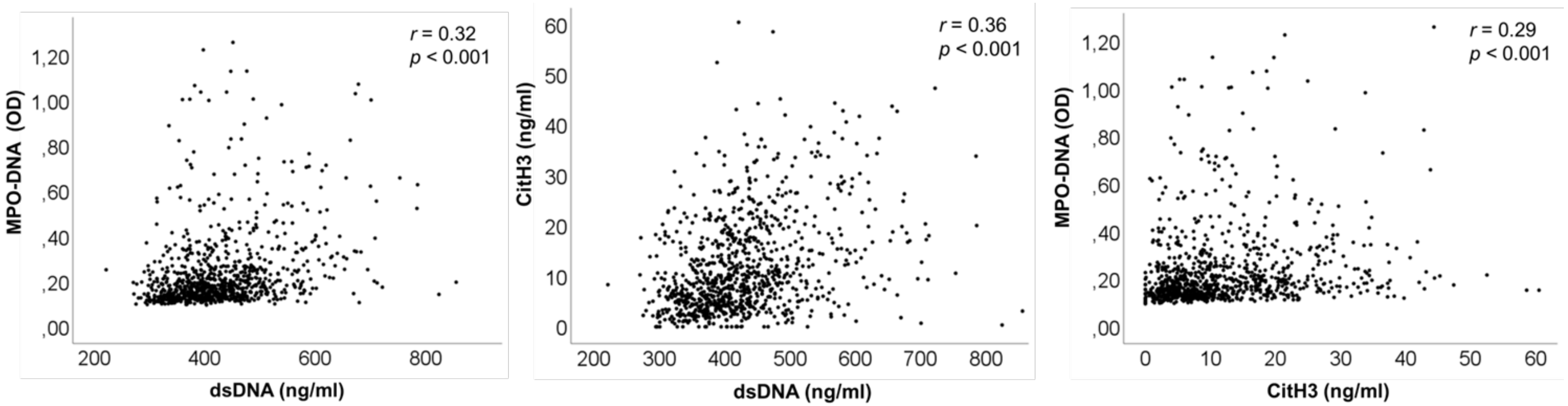

Figure S3. Levels of D-dimer and prothrombin fragment 1+2 (F1+2), according to below- or above-median levels of dsDNA. The *p*-values refer to Mann Whitney U tests. The height of each box represents the interquartile range (IQR), the horizontal line within each box is the median, and whiskers indicate 1.5 IQR.

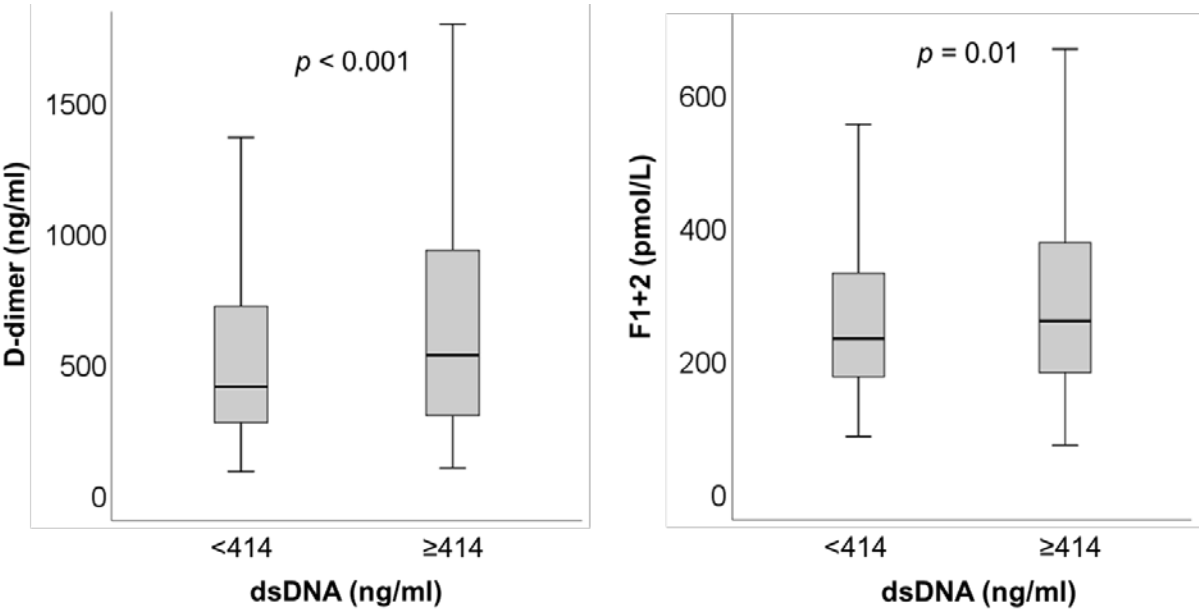

Table S1. Correlation table showing Spearman rho (r_s) analyses with 95% confidence intervals (CI) calculated using the Fisher Z transformation. Applying a Bonferroni correction for multiple testing, p-values <0.0024 are regarded as statistically significant.

		dsDNA	MPO-DNA	CitH3	Leukocyte count	Peak TnT	NT-proBNP	D-dimer	F1+2
dsDNA	r_s		0.32	0.36	0.22	0.17	0.19	0.17	0.08
	p	-	**<0.001**	**<0.001**	**<0.001**	**<0.001**	**<0.001**	**<0.001**	**0.021**
	n		956	955	952	956	926	950	950
	95% CI		0.26 to 0.38	0.30 to 0.41	0.16 to 0.28	0.11 to 0.23	0.13 to 0.25	0.11 to 0.23	0.01 to 0.14
MPO-DNA	r_s	0.32		0.29	0.18	0.12	-0.04	-0.02	-0.06
	p	**<0.001**	-	**<0.001**	**<0.001**	**<0.001**	0.255	0.552	0.068
	n	956		955	952	956	926	950	950
	95% CI	0.26 to 0.38		0.30 to 0.35	0.12 to 0.24	0.06 to 0.18	-0.10 to 0.03	-0.08 to 0.04	-0.12 to 0.00
CitH3	r_s	0.36	0.29		0.092	-0.02	0.10	-0.02	-0.05
	p	**<0.001**	**<0.001**	-	0.005	0.594	**0.001**	0.540	0.094
	n	955	955		951	955	925	949	949
	95% CI	0.30 to 0.41	0.30 to 0.35		0.03 to 0.15	-0.08 to 0.05	0.04 to 0.17	-0.08 to 0.04	-0.12 to 0.01

Table S2. Baseline characteristics of the study population according to above- or below-median levels of MPO-DNA and CitH3.

	MPO-DNA			CitH3		
	< median	≥ median	*p**	< median	≥ median	*p**
Age, mean (range)	62.0 (32-94)	59.5 (24-91)	**0.001**	60.9 (24-90)	60.6 (32-94)	0.675
Male sex	374 (77.9)	393 (82.6)	0.071	370 (77.4)	396 (83.0)	**0.029**
Current smoking	219 (45.6)	231 (48.5)	0.368	247 (51.7)	203 (42.6)	**0.005**
Hypertension	178 (37.1)	148 (31.1)	0.051	166 (34.7)	159 (33.3)	0.649
Diabetes	59 (12.3)	63 (13.2)	0.662	56 (11.7)	66 (13.8)	0.326
Previous CVD	117 (24.4)	111 (23.4)	0.715	104 (21.8)	123 (25.8)	0.139
Medication on admission						
- Single or DAPT	120 (25.0)	102 (21.4)	0.191	109 (22.8)	112 (23.5)	0.804
- Statins	125 (26.0)	90 (18.9)	**0.008**	110 (23.0)	105 (22.0)	0.711
- Beta-blockers	93 (19.4)	89 (18.7)	0.790	79 (16.5)	103 (21.6)	**0.046**
- ACEi/ARB	124 (25.8)	110 (23.1)	0.327	122 (25.5)	112 (23.5)	0.463
BMI, kg/m^2	26.7 (24.2, 29.2)	26.6 (24.3, 29.2)	0.850	26.9 (24.3, 29.2)	26.4 (24.3, 29.1)	0.466
Leukocyte count, x10^9/L	10.3 (8.4, 12.5)	11.3 (9.2, 14.1)	**<0.001**	10.4 (8.5, 12.9)	11.0 (8.9, 13.5)	**0.028**
Platelet count, x10^9/L	218 (187, 264)	222 (189, 266)	0.713	219 (185, 265)	222 (191, 265)	0.397
Total cholesterol, mmol/L	4.77 ±1.08	4.95 ±1.14	**0.014**	4.81 ±1.09	4.92 ±1.14	0.101
LDL-cholesterol, mmol/L	3.18 ±1.00	3.30 ±1.02	0.059	3.21 ±0.99	3.28 ±1.04	0.328
Fasting glucose, mmol/L	5.8 (5.2, 6.6)	5.8 (5.2, 6.7)	0.629	5.7 (5.2, 6.6)	5.8 (5.3, 6.7)	0.300
HbA_{1c}, %	6.0 (5.7, 6.3)	5.9 (5.6, 6.2)	0.054	5.9 (5.7, 6.2)	5.9 (5.6, 6.3)	0.304
NT-proBNP, ng/L	32 (12, 119)	28 (9, 118)	0.234	26 (10, 108)	39 (11, 129)	**0.016**
Peak TnT, ng/L	3390 (1575, 6538)	4400 (2018, 7840)	**0.001**	4055 (1715, 7538)	3760 (1720, 7110)	0.449
CRP, mg/L	12.5 (6.7, 29.2)	14.9 (7.5, 35.3)	0.128	12.4 (6.8, 27.3)	14.4 (7.5, 36.1)	0.080
LVEF ≤ 40 %	57 (15.6)	88 (23.6)	**0.006**	72 (19.6)	73 (19.7)	0.955
Prehospital thrombolysis	50 (10.4)	57 (12.0)	0.445	59 (12.3)	48 (10.1)	0.264
Anterior wall infarction	185 (38.5)	228 (47.9)	**0.003**	192 (40.2)	221 (46.3)	0.055
Time from symptom onset to blood sampling, hours	24 (19, 34)	23 (18, 32)	0.066	23 (18, 30)	24 (19, 37)	**0.002**

Values given as mean (±SD) or (range), median (25th, 75th percentiles) or numbers (%) as appropriate.
*p-value of Mann-Whitney U, Student's t, or Chi squared tests comparing groups with below- and above-median levels of MPO-DNA and CitH3.

DAPT: dual anti-platelet therapy
ACEi: angiotensin-converting enzyme inhibitor
ARB: angiotensin II receptor blocker
BMI: body mass index
LDL: low-density lipoprotein
HbA1c: hemoglobin A1c

Table S3. Baseline characteristics of the study population at inclusion according to composite endpoint and all-cause mortality groups.

	Composite endpoint			All-cause mortality		
	+ (n = 190)	- (n = 766)	*p**	+ (n = 76)	- (n = 880)	*p**
Age, mean (range)	63.9 (29-94)	60.0 (24-90)	**<0.001**	71.5 (34-94)	59.8 (24-90)	**<0.001**
Male gender	147 (77.4)	620 (80.9)	0.269	48 (63.2)	719 (81.7)	**<0.001**
Current smoking	87 (45.8)	363 (47.5)	0.670	33 (43.4)	417 (47.5)	0.495
Hypertension	74 (38.9)	252 (32.9)	0.115	38 (50.0)	288 (32.7)	**0.002**
Diabetes	32 (16.8)	90 (11.7)	0.060	18 (23.7)	104 (11.8)	**0.003**
Previous CVD	62 (32.6)	166 (21.7)	**0.002**	30 (39.5)	198 (22.5)	**0.001**
Medication on admission						
- Single or DAPT	63 (33.2)	159 (20.8)	**<0.001**	34 (44.7)	188 (21.4)	**<0.001**
- Statin	53 (27.9)	162 (21.1)	**0.046**	25 (32.9)	190 (21.6)	**0.024**
- Beta-blocker	47 (24.7)	135 (17.6)	**0.025**	26 (34.2)	156 (17.7)	**<0.001**
- ACEi/ARB	62 (32.6)	172 (22.5)	**0.003**	32 (42.1)	202 (23.0)	**<0.001**
BMI, kg/m^2	26.3 (24.2, 29.4)	26.6 (24.3, 29.1)	0.795	25.5 (23.9, 28.7)	26.7 (24.3, 29.2)	0.068
Leukocyte count, x 10^9/L	10.8 (8.4, 14.0)	10.7 (8.8, 13.1)	0.790	10.8 (8.4, 13.6)	10.7 (8.8, 13.2)	0.943
Platelet count, x 10^9/L	228 (191, 271)	219 (187, 263)	0.156	249 (200, 305)	219 (187, 263)	**<0.001**
Total cholesterol, mmol/L	4.71 ±1.21	4.90 ±1.09	**0.041**	4.40 ±1.27	4.90 ±1.10	**<0.001**
LDL-cholesterol, mmol/L	3.10 ±1.14	3.27 ±0.98	**0.043**	2.79 ±1.16	3.28 ±0.99	**<0.001**
Fasting glucose, mmol/L	5.9 (5.3, 6.9)	5.7 (5.2, 6.6)	**0.030**	6.1 (5.2, 7.5)	5.8 (5.2, 6.6)	**0.017**
HbA_{1c}, %	6.0 (5.7, 6.3)	5.9 (5.6, 6.2)	0.114	6.1 (5.8, 6.6)	5.9 (5.6, 6.2)	**0.001**
NT-proBNP, ng/L	50 (13, 201)	28 (10, 108)	**0.003**	102 (34, 336)	27 (10, 108)	**<0.001**
Peak TnT, ng/L	3790 (1410, 7458)	3875 (1810, 7183)	0.424	3960 (1485, 8515)	3845 (1753, 7140)	0.916
CRP, mg/L	14.8 (6.8, 35.5)	13.5 (7.1, 31.9)	0.968	16.4 (8.0, 74.0)	13.3 (7.0, 31.4)	0.243
LVEF ≤ 40 %	37 (26.4)	108 (18.1)	**0.025**	20 (35.1)	125 (18.4)	**0.002**
Prehospital thrombolysis	13 (6.8)	94 (12.3)	**0.034**	2 (2.6)	105 (11.9)	**0.014**
Anterior wall infarction	75 (39.5)	338 (44.1)	0.247	32 (42.1)	381 (43.3)	0.841
Time from symptom onset to blood sampling, hours	24 (19, 32)	24 (18, 33)	0.961	24.5 (20.0, 36.0)	24.0 (18.0, 32.0)	0.333

Values given as mean (±SD) or (range), median (25th, 75th percentiles) or numbers (%) as appropriate.
**p*-value of Mann-Whitney U, Student's t, or Chi squared tests comparing groups with (+) or without (-) clinical endpoint as appropriate

DAPT: dual anti-platelet therapy
ACEi: angiotensin-converting enzyme inhibitor
ARB: angiotensin II receptor blocker
BMI: body mass index
LDL: low-density lipoprotein
HbA1c: hemoglobin A1c

Table S4. Circulating levels of potential NETs-related markers in patients with (+) and without (-) a clinical endpoint.

	Primary composite endpoint			All-cause mortality		
	**+** (n = 190)	**-** (n = 766)	*p**	**+** (n = 76)	**-** (n = 880)	*p**
dsDNA, ng/ml	429 (371, 481)	412 (372, 466)	0.255	460 (407, 508)	411 (370, 466)	**<0.001**
MPO-DNA, OD	0.166 (0.138, 0.244)	0.181 (0.139, 0.267)	0.329	0.167 (0.140, 0.255)	0.178 (0.138, 0.261)	0.909
CitH3, ng/ml	8.92 (4.48, 16.52)	9.32 (4.91, 17.30)	0.459	10.25 (4.96, 17.32)	9.07 (4.83, 17.24)	0.598

Values are given as median (25th, 75th percentiles).

**p*-value of Mann-Whitney U test, comparing groups with (+) or without (-) a clinical endpoint.

Table S5. Crude/unadjusted and adjusted Cox regression analysis (Model 2) of the association between dsDNA and all-cause mortality. The adjusted multivariable model included age, sex, smoking, leukocyte count, platelet count, NT-proBNP, fasting glucose, CRP, D-dimer, previous CVD, peak TnT, prehospital thrombolysis, and time from symptom onset to blood sampling as potential covariates.

		Univariable analysis			Multivariable analysis 2 (*n* = 907)		
		Unadjusted			Adjusted		
	n	HR	95% CI	*p**	HR	95% CI	*p**
**dsDNA Q3 vs. Q1+2**	956	**2.03**	**1.12 to 3.66**	**0.019**	**1.88**	**1.01 to 3.49**	**0.045**
**dsDNA Q4 vs. Q1+2**	956	**3.36**	**1.95 to 5.78**	**<0.001**	**2.06**	**1.08 to 3.93**	**0.029**
Age	956	1.09	1.07 to 1.12	**<0.001**	1.09	1.07 to 1.13	**<0.001**
Male vs. female sex	956	0.40	0.25 to 0.64	**<0.001**	0.73	0.45 to 1.32	0.252
Current smoking (+/-)	956	0.87	0.55 to 1.36	0.535	1.99	1.11 to 3.24	**0.015**
Leukocyte count	953	1.03	1.00 to 1.06	**0.025**	0.98	0.87 to 1.02	0.634
Platelet count	952	1.01	1.00 to 1.01	**<0.001**	1.00	1.00 to 1.01	**0.019**
NT-proBNP	926	1.00	1.00 to 1.00	**<0.001**	1.00	1.00 to 1.00	0.376
Fasting glucose	947	1.17	1.05 to 1.29	**0.003**	1.24	1.11 to 1.40	**0.001**
CRP	955	1.01	1.00 to 1.01	**0.022**	1.00	1.00 to 1.01	0.587
D-dimer	950	1.00	1.00 to 1.00	**<0.001**	1.00	1.00 to 1.00	**0.038**
Previous CVD (+/-)	955	2.25	1.42 to 3.56	**0.001**	2.04	1.23 to 3.39	**0.006**
Peak TnT	956	1.03	0.99 to 1.07	0.150	1.03	0.99 to 1.07	0.220
Prehospital thrombolysis (+/-)	956	0.21	0.05 to 0.86	**0.030**	0.00	0.00 to 0.00	0.956
Time (symptom onset to blood sampling)	951	1.00	0.99 to 1.01	0.722	0.99	0.97 to 1.00	0.146

Hazard ratios per one unit increase in the explanatory variables, except where otherwise stated, e.g. presence/absence of categorical variables (+/-).
* *p*-value corresponding to Wald test.
